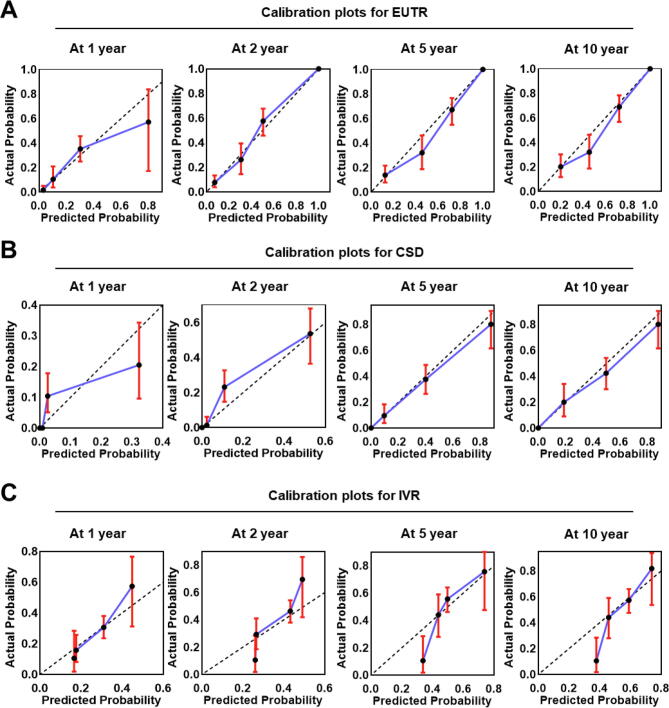# Site-specific Risk Stratification Models for Postoperative Recurrence and Survival Prediction in Patients with Upper Tract Urothelial Carcinoma Undergoing Radical Nephroureterectomy: Better Stratification for Adjuvant Therapy

**DOI:** 10.1016/j.euros.2022.05.004

**Published:** 2022-05-30

**Authors:** Makito Miyake, Kota Iida, Nobutaka Nishimura, Takashi Inoue, Hiroaki Matsumoto, Hideyasu Matsuyama, Yuya Fujiwara, Kazumasa Komura, Teruo Inamoto, Haruhito Azuma, Hiroaki Yasumoto, Hiroaki Shiina, Masaya Yonemori, Hideki Enokida, Masayuki Nakagawa, Hideo Fukuhara, Keiji Inoue, Takashi Yoshida, Hidefumi Kinoshita, Tadashi Matsuda, Tomomi Fujii, Kiyohide Fujimoto, Nishinihon Uro-oncology Extensive Collaboration Group

**Affiliations:** a Department of Urology, Nara Medical University, Kashihara, Nara, Japan; b Institute for Clinical and Translational Science, Nara Medical University, Kashihara, Nara, Japan; c Department of Urology, Graduate School of Medicine, Yamaguchi University, Ube, Yamaguchi, Japan; d Department of Urology, Osaka Medical and Pharmaceutical University, Takatsuki, Osaka, Japan; e Department of Urology, Shimane University School of Medicine, Izumo, Shimane, Japan; f Department of Urology, Graduate School of Medical and Dental Sciences, Kagoshima University, Sakuragaoka, Kagoshima, Japan; g Department of Urology, Kochi Medical School, Kohasu, Oko-cho, Nankoku-shi, Kochi, Japan; h Department of Urology and Andrology, Kansai Medical University, Osaka, Japan; i Department of Diagnostic Pathology, Nara Medical University, Kashihara, Nara, Japan

**Keywords:** Adjuvant therapy, Death, Nephroureterectomy, Prediction, Prognosis, Recurrence, Risk, Upper urinary tract carcinoma, Urinary tract, Urinary bladder neoplasm

## Abstract

**Background:**

Site-specific postoperative risk models for localized upper tract urothelial carcinoma (UTUC) are unavailable.

**Objective:**

To create specific risk models for renal pelvic urothelial carcinoma (RPUC) and ureteral urothelial carcinoma (UUC), and to compare the predictive accuracy with the overall UTUC risk model.

**Design, setting, and participants:**

A multi-institutional database retrospective study of 1917 UTUC patients who underwent radical nephroureterectomy (RNU) between 2000 and 2018 was conducted.

**Outcome measurements and statistical analysis:**

A multivariate hazard model was used to identify the prognostic factors for extraurinary tract recurrence (EUTR), cancer-specific death (CSD), and intravesical recurrence (IVR) after RNU. Patients were stratified into low-, intermediate-, high-, and highest-risk groups. External validation was performed to estimate a concordance index of the created risk models. We investigated whether our risk models could aid decision-making regarding adjuvant chemotherapy (AC) after RNU.

**Results and limitations:**

The UTUC risk models could stratify the risk of cumulative incidence of three endpoints. The RPUC- and UUC-specific risk models showed better stratification than the overall UTUC risk model for all the three endpoints, EUTR, CSD, and IVR (RPUC: concordance index, 0.719 vs 0.770, 0.714 vs 0.794, and 0.538 vs 0.569, respectively; UUC: 0.716 vs 0.767, 0.766 vs 0.809, and 0.553 vs 0.594, respectively). The UUC-specific risk model can identify the high- and highest-risk patients likely to benefit from AC after RNU. A major limitation was the potential selection bias owing to the retrospective nature of this study.

**Conclusions:**

We recommend using site-specific risk models instead of the overall UTUC risk model for better risk stratification and decision-making for AC after RNU.

**Patient summary:**

Upper tract urothelial carcinoma comprises renal pelvic and ureteral carcinomas. We recommend using site-specific risk models instead of the overall upper tract urothelial carcinoma risk model in risk prediction and decision-making for adjuvant therapy after radical surgery.

## Take Home Message

There have been limited reports of postoperative risk stratification using predictive tools in patients with upper tract urothelial carcinoma. Renal pelvic urothelial carcinoma and ureteral urothelial carcinoma have anatomical, biological, and molecular differences, including in the immunological profile and clinical behavior. We recommend using site-specific risk models instead of the overall upper urinary tract carcinoma risk model for better risk stratification and decision-making for adjuvant therapy after radical nephroureterectomy.

## 1 Introduction

Urothelial carcinoma (UC) arises in the renal pelvis, ureters, bladder, or urethra. Upper tract UC (UTUC), including renal pelvic UC (RPUC) and ureteral UC (UUC), accounts for 5−10% of UCs [1]. Radical nephroureterectomy (RNU) with complete removal of the ureteral orifice remains the standard surgical treatment for localized UTUC [2]. UTUC is aggressive, especially in high-grade and high-stage tumors, and has a poor clinical outcome, with a 5-yr cancer-specific survival rate of 66–80% [1], [3], [4]. Currently, neoadjuvant chemotherapy (NAC) and/or adjuvant chemotherapy (AC) is used to improve the prognosis of UTUC. Studies have demonstrated that RNU with both NAC and AC provides better survival than RNU alone [5]. In the 2021 European Association of Urology guidelines on UTUC, the evidence level of AC was positive level 1b, and platinum-based AC was strongly recommended for patients with pT2 ≤ UTUC or N+ disease, while the evidence level for NAC remains level 2 [6].

In the Peri-Operative chemotherapy versus sUrveillance in upper Tract urothelial cancer (POUT) trial, a phase 3 prospective randomized trial, patients with pT2-pT4 N (any) M0 or pT (any) N1–3 M0 UTUC benefited from gemcitabine-platinum combination AC initiated within 90 d after RNU [7]. A subgroup analysis indicated a greater survival benefit from AC in the pN0, pT3/4, and cisplatin regimen groups than in the N+, pT2, and carboplatin regimen groups. The subgroup analyses indicated that not all high-risk populations can benefit from time-consuming and invasive systemic chemotherapy; therefore, clinicians must consider the risk of overtreatment. A more accurate postoperative risk stratification model based on real-world data is required for appropriate decision-making and patient consultation for adjuvant therapy.

In managing UC of the bladder, several clinicopathological parameter–based risk scoring tables are widely used for predicting the short- and long-term probabilities of oncological events [8], [9], [10]. Owing to the rarity and heterogeneous biology and behavior of UTUC, there have been limited reports of patient risk stratification using predictive tools [11]. RPUC and UUC have many similarities and are generally considered collectively as UTUC disease sets. However, there are anatomical, biological, and molecular differences, including in the immunological profile and clinical behavior, suggesting two distinct urothelium-derived malignancies [12]. To the best of our knowledge, no study has been conducted to confirm the usefulness of RPUC- and UUC-specific risk stratification models.

This study aimed to develop site-specific risk stratification models for predicting postoperative extraurinary tract recurrence (EUTR), cancer-specific death (CSD), and intravesical recurrence (IVR) in patients with RPUC or UUC undergoing RNU. Subsequently, the models were externally validated to assess their applicability in patient selection for adjuvant therapy.

## 2 Patients and methods

### 2.1 Study cohorts of UTUC and data collection

This retrospective multicenter study was approved by the ethics committee of each participating institute (reference ID: 1298, 1958, 2891, H30-048, and 2018036) of the Nishinihon Uro-Oncology Extensive Collaboration Group framework. Informed consent was obtained from the participants or bereaved families through posters and/or websites using the opt-out method [13].

**Fig. 1:**
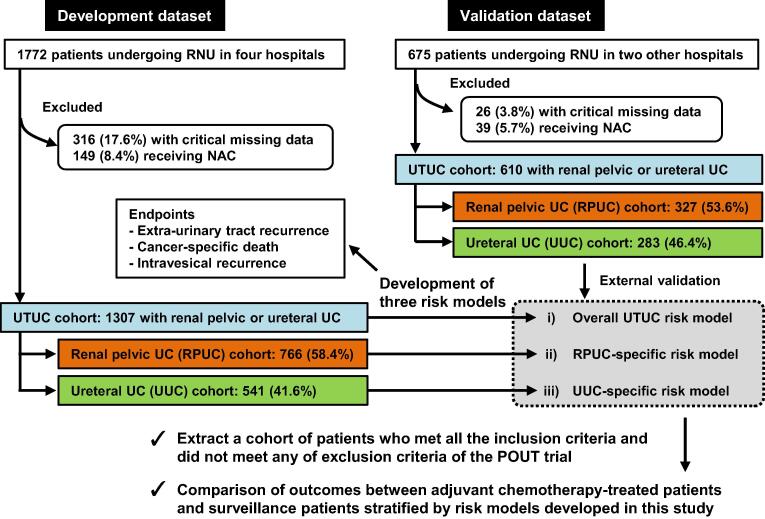
Flow chart for creation of the patient cohort dataset. This study used two independent datasets: development and validation. From the original datasets, the cohort excluded patients who were treated with neoadjuvant chemotherapy (NAC) or had critical missing data. Based on the factor coefficients of the multivariate Fine and Gray subdistribution hazard models, three risk stratification models for extraurinary tract recurrence, cancer-specific death, and intravesical recurrence were developed. The models were validated externally using a validation dataset. Additionally, we investigated whether the developed risk models could provide better stratification to select patients who are likely to benefit from adjuvant chemotherapy. NAC = neoadjuvant chemotherapy; POUT = Peri-Operative chemotherapy versus sUrveillance in upper Tract urothelial cancer; RNU = radical nephroureterectomy; UC = urothelial carcinoma; UTUC = upper tract urothelial carcinoma.

We reviewed the medical charts of 2447 consecutive patients with UTUC who underwent RNU and were diagnosed with UC between 2000 and 2018 at six institutions across Western Japan (Fig. 1). Of the 2447 patients, 342 (14%) were excluded because of critical missing data. One of the study aims was to develop risk stratification models for AC; therefore, 188 patients (7.7%) who received NAC were excluded. The methods used for lymph node dissection was inconsistent among surgeons and hospitals, and changed over time. In general, a template-based dissection that was dependent on tumor location [14] was performed in our collaborative academic hospitals for UTUC patients with suspected tumors ≥T2 or clinically node-positive tumors. The 1307 patients initially registered from four institutions were used as the development dataset, and another independent dataset of 610 patients, additionally registered from two institutions, was used for external validation.

### 2.2 Follow-up and endpoints

The standard protocol was generally used for the follow-up after RNU: cystoscopy, urinary cytology, and abdominopelvic and chest computed tomography (CT) are performed every 3 mo for 2 yr, every 6 mo until 5 yr, and then yearly. This study evaluated three endpoints: EUTR, CSD, and IVR. EUTR was defined as any recurrence, excluding IVR and contralateral UTUC. While IVR is generally considered non–life threatening, EUTR includes life-threatening events such as local recurrence in soft tissue, regional lymph node involvement, and metastatic disease. Other-cause deaths before EUTR, CSD, and IVR were analyzed as competing risks. Patients who were alive without events were censored at the dates of the last follow-up cystoscopy for IVR, last imaging examination for EUTR, and last visit for CSD.

### 2.3 Identification of prognostic factors and development of new risk tables

To develop multiple risk stratification models—“the Japanese NIshinihon uro-onCology Extensive collaboration group (J-NICE) risk models”, first, univariate and multivariate Fine and Gray subdistribution hazard models were applied for all endpoints to assess the prognostic impact of clinicopathological factors in our cohort [15]. Second, the best combination of variable subsets was selected from all the variable combinations according to the Akaike information criterion [16]. Third, the score allocated to each selected prognostic factor was derived from rounding up regression coefficients of the final multivariate models. Fourth, the total score corresponding to a given patient’s factors was summed up. Fifth, patients were divided into four groups according to their total score. For each group, cumulative incidence estimates of the probabilities at three time points (2, 5, and 10 yr) were calculated, with 95% set as the confidence interval (CI).

### 2.4 External validation using an independent dataset

External validation was conducted using an independent dataset of 610 patients with UTUC treated in two other hospitals. The patients were stratified into four groups according to the J-NICE risk table. The model performance was assessed with respect to calibration and discrimination. The calibration plot was graphically generated to examine the relationship between the observed cumulative incidence estimates and the predicted probabilities for each risk group. The stratification ability (discrimination) for events in the J-NICE risk tables was evaluated using the concordance index (c-index) for the three endpoints.

### 2.5 Statistical analysis

The statistical software EZR, which is based on the open-source R statistical software (version 3.6.1; R Foundation for Statistical Computing, Vienna, Austria), and PRISM software version 9 (GraphPad Software, Inc., San Diego, CA, USA) were used for statistical analyses and data visualization, respectively. We used Mann-Whitney *U* test for continuous variables and the chi-square test to compare the proportions of categorical variables between the groups. Survival packages were used to calculate c-index for our study. Statistical significance was set at *p* < 0.05.

## 3 Results

### 3.1 Development of risk scoring tables for three endpoints

Clinicopathological characteristics of the patients are presented in Supplementary Table 1. Among the 1917 (total) patients, the pathological N category data were available for 1319 (72%) patients undergoing lymph node dissection. Pathologically positive lymph node involvement was detected in 172 (9.0%) patients. None of the patients underwent early postoperative intravesical chemotherapy. In the development dataset (*n* = 1307), during follow-up after RNU (median, 37.1 mo; interquartile range [IQR] 16.6−69.0), 362 (27.7%) and 470 (36.0%) patients experienced EUTR and IVR, respectively. A total of 250 (19.1%) patients died of UC and 87 (6.7%) died of other causes (competing risks). Prognostic factors for each endpoint in the UTUC development cohort (*n* = 1307), RPUC development cohort (*n* = 766), and UUC development cohort (*n* = 541) were identified using univariate and subsequent multivariate analyses (Supplementary Tables 2−4). Each patient’s score was calculated based on the factor coefficients in the multivariate model, and patients were divided into four groups according to the total J-NICE score: low-, intermediate-, high-, or highest-risk group (Table 1). Supplementary Table 5 shows the actual probabilities after RNU at 2, 5, and 10 yr and their 95% CIs according to patient risk.

**Table 1:** The J-NICE risk tables for calculating risk scores for extra-urinary tract recurrence, cancer-specific death, and intravesical recurrence in the UTUC patients undergoing radical surgery^a^

Factors	Overall UTUC risk model			RPUC-specific risk model			UUC-specific risk model		
	EUTR (6)	CSD (5)	IVR (6)	EUTR (4)	CSD (3)	IVR (4)	EUTR (3)	CSD (6)	IVR (5)
Sex									
Male			1			1		0	
Female			0			0		1	
Location of main tumor									
Renal pelvis	0	0	0						
Upper ureter	0	0	0						
Middle ureter	1	1	1						
Lower ureter	1	0	1						
Multifocality									
Solitary			0			0			0
Multiple			1			1			1
Hydronephrosis									
No			1						1
Yes			0						0
Baseline hemoglobin									
≥ LLN								0	0
<LLN								2	1
Baseline NLR									
≤3.0	0	0	0				0	0	0
>3.0	1	1	1				1	1	1
Clinical N category									
N0	0	0		0	0				
N+	2	2		3	3				
Pathological T category									
Ta/Tis	0	0	2	0	0	2	0	0	1
T1	1	1	2	1	2	2	0	0	1
T2	2	2	2	2	2	2	1	1	1
T3	3	3	1	3	3	2	2	2	0
T4	4	4	0	4	4	0	5	3	0
Tumor grade (WHO 2004)									
Low grade	0			0		0			
High grade	1			2		1			
Carcinoma in situ									
Negative								1	
Positive								0	
Lymphovascular invasion									
No	0	0		0	0		0	0	
Yes	2	2		2	2		3	2	
Total scores	0–11	0–10	0–7	0–11	0–9	0–5	0–9	0–10	0–5
Risk stratification									
Low-risk	0–2	0–1	0–1	0–2	0–2	0–1	0–1	0–1	0
Intermediate-risk	3–5	2–4	2–3	3–5	3–4	2–3	2–4	2–4	1
High-risk	6–8	5–7	4–5	6–8	5–6	4	5–6	5–7	2–3
Highest-risk	9–11	8–10	6––7	9-11	7–9	5	8–9	8–10	4–5

CSD = cancer-specific death; EUTR = extraurinary tract recurrence; IVR = intravesical recurrence; J-NICE = Japanese NIshinihon uro-onCology Extensive collaboration group; LLN = lower limit of the normal; NLR = neutrophil lymphocyte rate; RPUC = renal pelvic urothelial cancer; UTUC = upper urinary tract cancer; UUC = ureteral urothelial carcinoma; WHO = World Health Organization.

a The allocated scores were determined by rounding up regression coefficients shown in Supplementary Tables 2–4. For example, prognostic factors with regression coefficients >0 and ≤1 were allocated 1 point, prognostic factors with regression coefficients >1 and ≤2 were allocated 2 points, prognostic factors with regression coefficients >2 and ≤3 were allocated 3 points, and prognostic factors with regression coefficients >3 were allocated 4 points. On the contrary, 1 or 2 points were allocated to the counterparts when regression coefficients were >–1 and <0 or >–2 and <–1, respectively.

### 3.2 External validation using an independent dataset

**Fig. 2:**
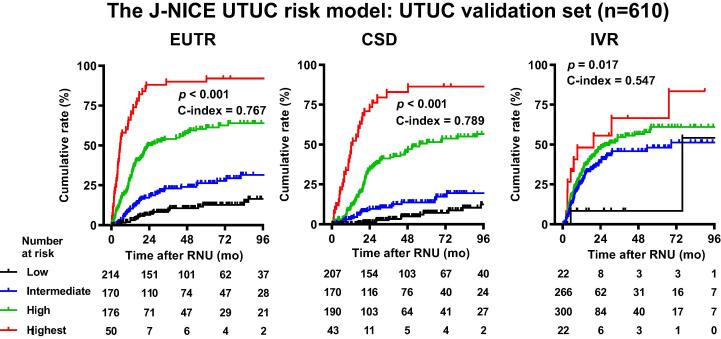
External validation of the J-NICE UTUC risk model. The times to extraurinary recurrence (EUTR), cancer-specific death (CSD), and intravesical recurrence (IVR) were stratified according to J-NICE risk models. The UTUC risk model was applied to 610 UTUC patients in the validation dataset. The patients were divided into four groups according to their total score, as shown in Table 1. The *p* values and the bias-corrected c-indices for the scoring models for EUTR, CSD, and IVR are shown in each survival plot. J-NICE = Japanese NIshinihon Uro-onCology Extensive Collaboration Group; RNU = radical nephroureterectomy; UTUC = upper urinary tract carcinoma.

We conducted external validation on an independent dataset of patients with UTUC. The development and validation sets showed no significant difference in the location of the main tumor and pathological T category, whereas clinical N category, tumor grade, baseline hemoglobin, and baseline neutrophil lymphocyte ratio (NLR) showed significant differences (Supplementary Table 1). In the validation dataset (*n* = 610), during follow-up after RNU (median, 34.4 mo; IQR 16.8−71.3), 203 (33.3%) and 245 (40.2%) patients experienced EUTR and IVR, respectively. In all, 150 (24.6%) patients died of UC and 74 (12.1%) died of other causes (competing risks). The J-NICE overall UTUC risk models could stratify the risks of cumulative incidence of EUTR, CSD, and IVR for the development (data not shown) and validation datasets (Fig. 2).

**Fig. 3:**
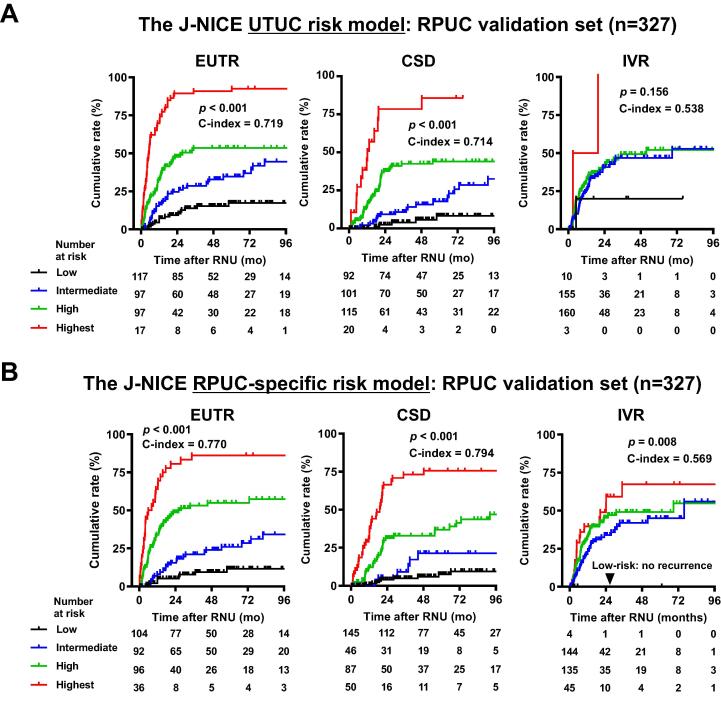
External validation of the J-NICE RPUC-specific risk model: comparison with the UTUC risk model. The times to extraurinary recurrence (EUTR), cancer-specific death (CSD), and intravesical recurrence (IVR) were stratified according to the (A) J-NICE UTUC risk model and (B) RPUC-specific risk model. Risk models were applied to the 327 patients with RPUC in the validation dataset. The patients were divided into four groups according to their total score, as shown in Table 1. The *p* values and the bias-corrected c-indices for the scoring models for EUTR, CSD, and IVR are shown in each survival plot. J-NICE = Japanese NIshinihon Uro-onCology Extensive Collaboration Group; RNU = radical nephroureterectomy; RPUC = renal pelvic urothelial carcinoma; UTUC = upper urinary tract carcinoma.

**Fig. 4:**
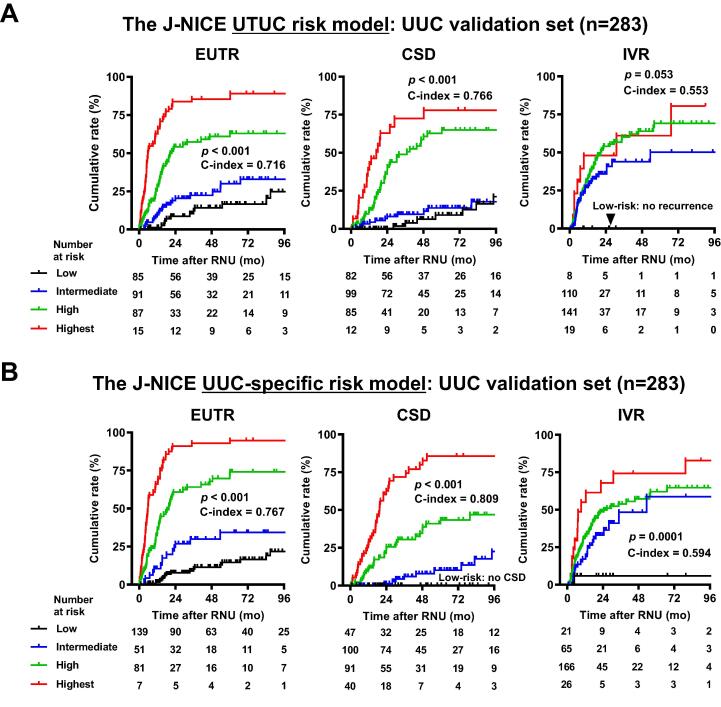
External validation of the J-NICE UUC-specific risk model: comparison with the UTUC risk model. The times to extraurinary recurrence (EUTR), cancer-specific death (CSD), and intravesical recurrence (IVR) were stratified according to the (A) J-NICE UTUC risk model and (B) the UUC-specific risk model. The risk models were applied to 283 patients with UUC in the validation dataset. The patients were divided into four groups according to their total score, as shown in Table 1. The *p* values and the bias-corrected c-indices for the scoring models for EUTR, CSD, and IVR are shown in each survival plot. J-NICE = Japanese NIshinihon Uro-onCology Extensive Collaboration Group; RNU = radical nephroureterectomy; UTUC = upper urinary tract carcinoma; UUC = ureteral urothelial carcinoma.

**Supplementary figure 1:**
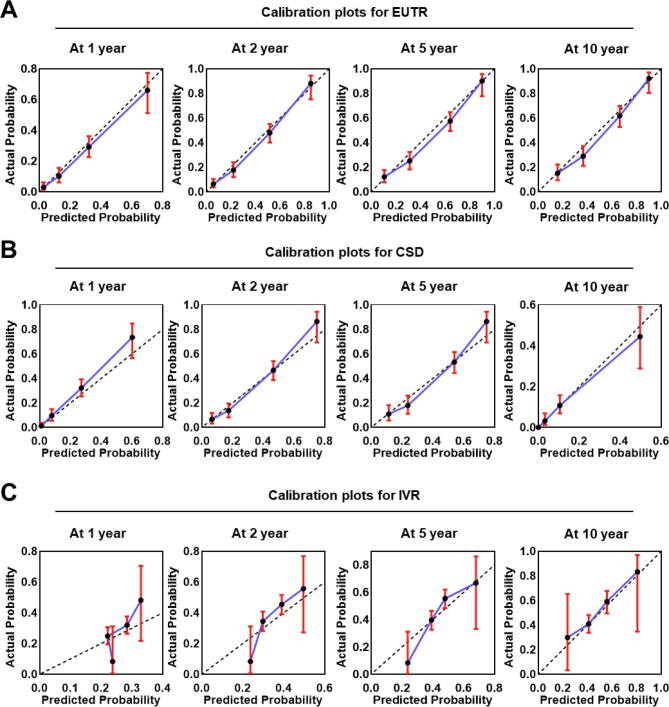


**Supplementary figure 2:**
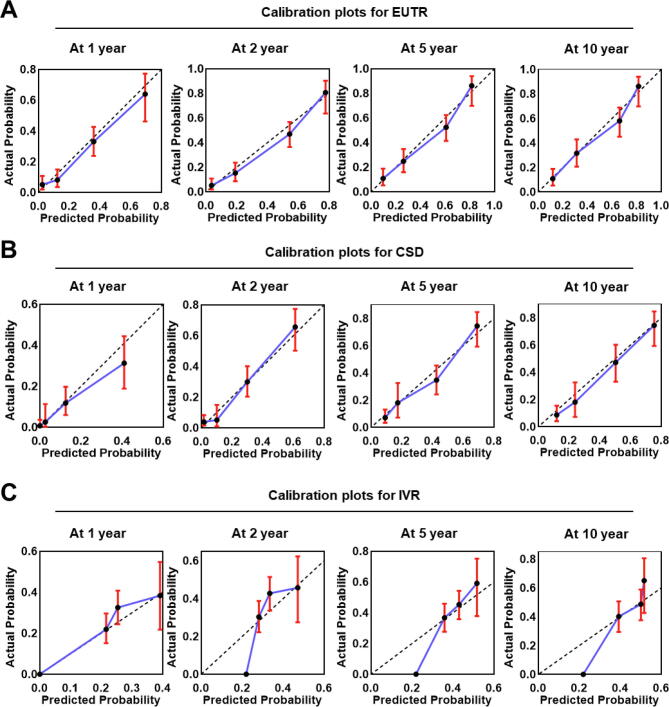


Next, we investigated whether site-specific risk models showed better stratification of the cumulative incidence risk than the overall UTUC risk model. A comparison of the accuracy for predicting the outcomes in the RPUC validation set (*n* = 327) between the overall UTUC risk model and each specific risk model revealed better stratification for all three endpoints: EUTR (c-index, 0.719 vs 0.770), CSD (0.714 vs 0.794), and IVR (0.538 vs 0.569) in the RPUC model (Fig. 3), and EUTR (0.716 vs 0.767), CSD (0.66 vs 0.809), and IVR (0.553 vs 0.594) in the UUC model (Fig. 4). Calibration plots of the relationships between the actual 1-, 2-, 5-, and 10-yr cumulative incidences for three endpoints and the predicted probabilities for each risk group using the external validation set are shown in the Supplementary Figures 1−3, demonstrating that the predicted EUTR and CSD rates from the risk table correlated closely with the actual observations of survival in the external validation set. Thus, the external validation suggests that the site-specific risk models may better fit the real-world clinical practice of UTUC risk assessment than the overall UTUC risk model.

### 3.3 Identification of patients who are likely to benefit from AC

**Fig. 5:**
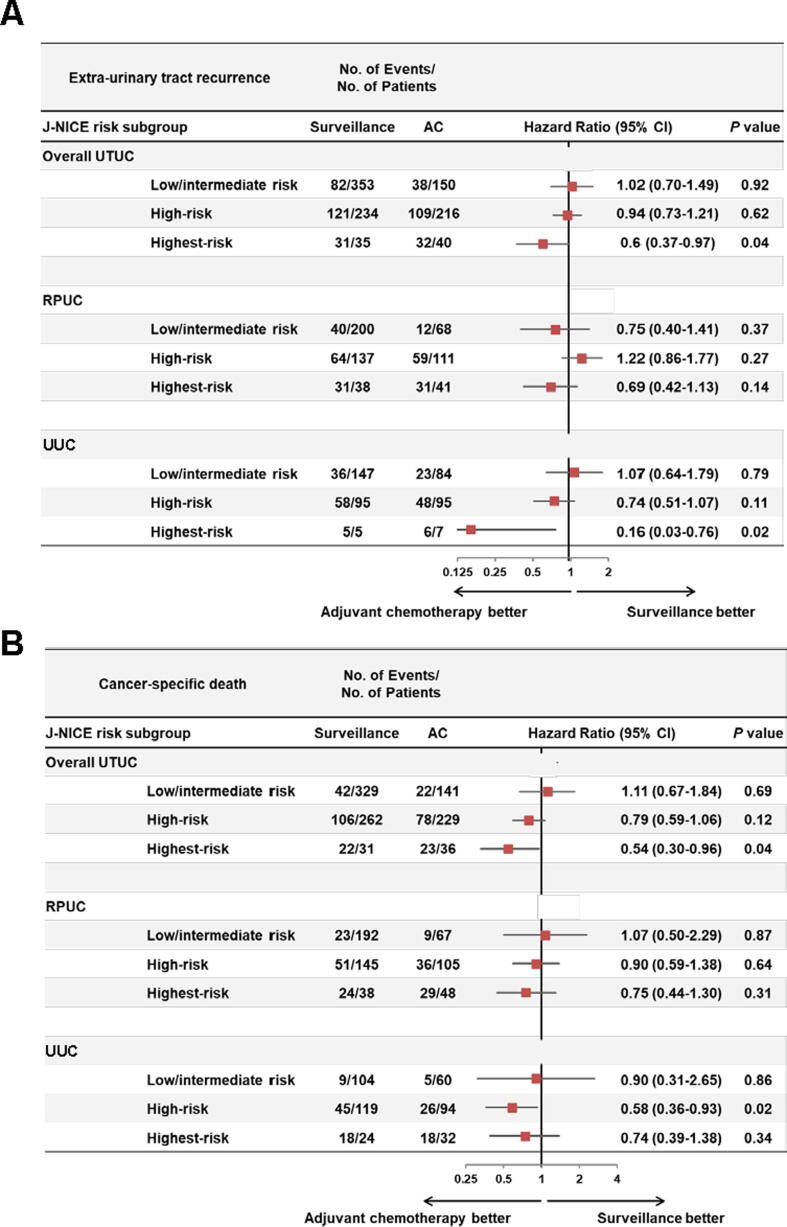
An analysis of extraurinary tract recurrence and cancer-specific death in risk subgroups stratified the J-NICE risk models in the POUT-eligible patients. The POUT-eligible patients were defined as those who met all the inclusion criteria and none of the exclusion criteria of the POUT trial. A total of 1028 POUT-eligible patients were stratified into low-, intermediate-, high-, and highest-risk groups according to the total score, as shown in Table 1. The Fine and Gray subdistribution hazard models were used to calculate HR and 95% CI. AC = adjuvant chemotherapy; CI = confidence interval; HR = hazard ratio; J-NICE = Japanese NIshinihon Uro-onCology Extensive Collaboration Group; POUT = Peri-Operative chemotherapy versus sUrveillance in upper Tract urothelial cancer; RPUC = RPUC = renal pelvic urothelial carcinoma; UTUC = upper urinary tract carcinoma; UUC = ureteral urothelial carcinoma.

To identify patients at a high-risk of UTUC who are likely to benefit from AC after RNU, we extracted a cohort of patients who met all the inclusion criteria and none of the exclusion criteria of the POUT trial referring to the trial identifier NCT01993979 of ClinicalTrials.gov [7]. We paid special attention to pT category, pN category, variant histology, hematological profile, liver function tests, postoperative renal function (glomerular filtration rate), and World Health Organization performance status. Of 1917 patients, 1028 (53.6%) including 595 with RPUC and 433 with UUC were defined as the POUT-eligible patients. We compared the outcomes between AC-treated and surveillance patients among 1028 POUT-eligible patients stratified by the J-NICE risk models. Figure 5 summarizes the subgroup analyses for EUTR and CSD with patients stratified by the J-NICE risk models, revealing that the UUC-specific risk model could help stratify high- and highest-risk patients who are likely to benefit from AC after RNU (Fig. 5). AC provided significant benefit for EUTR and CSD in the highest-risk group stratified by the overall UTUC risk model.

## 4 Discussion

Much effort has been made to create a universal risk stratification tool for the management of UTUC that could aid in identifying candidates for perioperative therapy, such as neoadjuvant or adjuvant therapy. The novelty of our study includes the creation of RPUC- and UUC-specific risk models, separately from the overall UTUC risk model. Almost all currently available risk stratification models were created and developed by incorporating both RPUC and UUC into a single risk model [11]. Bladder UC, RPUC, and UUC commonly arise from the urothelial mucosa and have many similarities, but also have many differences [12], [17], [18]. We previously demonstrated that the immunological profile of the tumor microenvironment differs significantly between UCs arising from the renal pelvis and ureter [12]. This led us to consider bladder UC, RPUC, and UUC as disparate triplets biologically and clinically.

To create the risk scoring tables, we evaluated several patient- and tumor-related factors that could influence outcomes and prognosis. Common risk factors in the RPUC- and UUC-specific risk models included sex, multifocality, pathological T category, and lymphovascular invasion. Notably, there were factors that were not common to the two risk models. For example, tumor grade and clinical N category are included in the RPUC-specific but not in the UUC-specific risk model. The presence of hydronephrosis, baseline hemoglobin, baseline NLR, and presence of carcinoma in situ were included in the UUC-specific but not in the RPUC-specific risk model. This discrepancy suggests that a site-specific risk model could provide better stratification than the overall UTUC risk model. The comparison using the external validation dataset revealed that both site-specific risk models showed higher c-indices on all endpoints and provide better risk stratification than the overall UTUC risk model (Fig. 3, Fig. 4), suggesting that our risk model better fits clinical practice of UTUC risk assessment. All six noncommon factors—tumor grade [4], [19], clinical N category [19], presence of hydronephrosis [20], baseline hemoglobin [21], baseline NLR [22], and presence of CIS [23]—have been reported as significant risk factors for patients with UTUC undergoing RNU. Although these factors contribute to oncological outcomes in overall UTUC cohorts, the weighting may be unbalanced between two subsets. The EORTC [8] and CUETO [9] risk tables are widely used in the clinical management of non–muscle-invasive bladder cancer. Given that no risk stratification table has widely been accepted yet in the management of UTUC [11], robustness of risk stratification should be improved gradually to suit real-world clinical practice; this indicates the need for more accurate and user-friendly risk stratification models. As expected, the stratification ability (discrimination) for events in the site-specific J-NICE risk tables work better than overall UTUC risk tables based on the c-index for the three endpoints in the external validation. We believe that accurate and user-friendly J-NICE risk tables will be used more widely in the clinical practice of UTUC after sufficient external validation.

Although the POUT trial showed a great impact of AC on disease-free and metastasis-free survival, chemotherapy-induced acute toxicity and a transient negative impact on patient-reported quality of life have been reported [7]. Zhang et al [24] reported that patient-reported anxiety and depression increased during AC in patients with muscle-invasive bladder cancer after radical cystectomy. The negative effects of AC should not be ignored. Owing to the heterogeneous nature of UTUC and the overtreatment risk of perioperative chemotherapy, decision-making for proper therapeutic options is challenging. In this study, we expected that our site-specific risk models could stratify the POUT-eligible patients who are likely to benefit from AC. Theoretically, AC could improve clinical outcomes by reducing the viability of residual and circulating cancer cells after RNU. Our findings prove that patients with J-NICE high- and highest-risk UTUC, especially those with UUC, have possible residual and circulating cancer cells (Fig. 5). Patients with low- and intermediate-risk UTUC might be surveyed postoperatively without AC to avoid treatment-related adverse events and reduce medical costs. The latest evidence demonstrates that RNU with AC for advanced UTUC provides a survival benefit compared with RNU alone [5], [6], [25]. A recent clinical trial, Checkmate 274, demonstrated the positive role of adjuvant nivolumab in high-risk muscle-invasive UC after radical surgery [26]. Of the enrolled patients, 560 (79%) had bladder cancer, 96 (14%) had RPUC, and 53 (7.5%) had UUC. The subgroup analysis demonstrated that adjuvant nivolumab provided a disease-free survival benefit only in bladder cancer (hazard ratio [HR], 0.62; 95% CI 0.49−0.78), while no significant benefit was observed in RPUC (HR 1.16; 95% CI 0.63−2.13) and UUC (HR 1.55, 95% CI 0.70−3.45). Platinum-based chemotherapy remains the gold-standard adjuvant option for high-risk UTUC.

This study has several limitations. First, the retrospective study design has an inherent potential for selection bias, and the decision criteria for the implementation of NAC/AC, chemotherapy regimen, timing of changing the treatment, and interval of radiographic evaluation were dependent on the institutional protocol and the physician’s discretion. The cohort was derived from multiple institutions, which may have introduced inconsistencies in surgical skills, timing of ureteral ligation, clinical interpretation, and pathological diagnoses. Second, the treatment strategy; modality, especially approval of gemcitabine plus platinum combination chemotherapy and advent of immune checkpoint inhibitors; and surgical skill change over time may influence outcomes. A substantial population (28%) of patients did not undergo lymph node dissection. Moreover, the number of dissected lymph nodes and the data pertaining to positive surgical margins were not available. Third, the cohort did not include patients undergoing kidney-sparing treatment, such as segmental nephroureterectomy and endoscopic laser ablation, which are currently recommended for low-grade, solitary, and small tumors. Fourth, statistical power may be limited because of the small number of patients and events in some subgroups. Fifth, this study did not analyze molecular biomarkers such as PD-1 or PD-L1 immunostaining and other possible biological factors, which may have strengthened the predictive power of our risk models. Further research is required to consolidate our findings and confirm the molecular aspects influencing the clinical effects of AC.

## 5 Conclusions

To the best of our knowledge, this is the first study to develop an RPUC-specific and a UUC-specific risk model, separately from the overall UTUC risk model. Future external validation with more extensive and diversified patient cohorts is vital to confirm real-world clinical impact.

  ***Author contributions*:** Makito Miyake had full access to all the data in the study and takes responsibility for the integrity of the data and the accuracy of the data analysis.

*Study concept and design:* Miyake, Fujimoto.

*Acquisition of data:* Nishimura, Matsumoto, Fujiwara, Komura, Inamoto, Yasumoto, Shiina, Yonemori, Enokida, Fukuhara, Yoshida, Kinoshita.

*Analysis and interpretation of data:* Iida, T. Inoue, Fujii.

*Drafting of the manuscript:* Miyake.

*Critical revision of the manuscript for important intellectual content:* Matsuyama, Fujimoto.

*Statistical analysis:* Iida, T. Inoue.

*Obtaining funding:* Miyake, Fujimoto.

*Administrative, technical, or material support:* Fujii.

*Supervision:* Azuma, Matsuda, K. Inoue, Nakagawa.

*Other:* None.

  ***Financial disclosures:*** Makito Miyake certifies that all conflicts of interest, including specific financial interests and relationships and affiliations relevant to the subject matter or materials discussed in the manuscript (eg, employment/affiliation, grants or funding, consultancies, honoraria, stock ownership or options, expert testimony, royalties, or patents filed, received, or pending), are the following: None.

  ***Funding/Support and role of the sponsor*:** This research was supported by Japan Society for the Promotion of Science (JSPS) KAKENHI grant numbers 1592057 (Kiyohide Fujimoto), 16K20159 (Makito Miyake), and 26861290 (Kiyohide Fujimoto), and Fiscal Years 2015–2016 Nara Medical University Grant-in-Aid for the Collaborative Research Projects (Kiyohide Fujimoto and Makito Miyake).

  ***Data sharing*:** Data are available for bona fide researchers who request it from the authors.

## Figures and Tables

**Supplementary figure 3:**