# Supplementary material for: Site-specific Risk Stratification Models for Postoperative Recurrence and Survival Prediction in Patients with Upper Tract Urothelial Carcinoma Undergoing Radical Nephroureterectomy: Better Stratification for Adjuvant Therapy
Source: Eur Urol Open Sci. 2022 May 30;41:95–104. doi: 10.1016/j.euros.2022.05.004 (PMC9257658; doi:10.1016/j.euros.2022.05.004)
Supplement: Supplementary data 1 [file mmc1.docx]

| **Supplementary Table 1. Clinicopathological characteristics of 1917 patients with UTUC undergoing RNU and comparison between the developmental set and validation set.** | | | | | |
| --- | --- | --- | --- | --- | --- |
| Variables |  | Total | Development dataset | Validation dataset | *p value* |
| N |  | 1917 (100%) | 1307 (100%) | 610 (100%) | - |
| Age at RNU, median (IQR) |  | 73 (66-79) | 73 (65-79) | 73 (67-79) | 0.03 |
| Sex | Male | 1355 (71%) | 929 (71%) | 426 (70%) | 0.59 |
|  | Female | 562 (29%) | 378 (29%) | 184 (30%) |  |
| Location of main tumour | Renal pelvis | 1093 (57%) | 766 (59%) | 327 (54%) | 0.12 |
|  | Upper ureter | 189 (9.9%) | 126 (9.6%) | 63 (10%) |  |
|  | Middle ureter | 254 (13%) | 159 (12%) | 95 (16%) |  |
|  | Lower ureter | 381 (20%) | 256 (20%) | 125 (21%) |  |
| Multifocality | Solitary | 1571 (82%) | 1085 (83%) | 486 (80%) | 0.09 |
|  | Multiple | 346 (18%) | 222 (17%) | 124 (20%) |  |
| Hydronephrosis | No | 929 (49%) | 639 (49%) | 290 (48%) | 0.59 |
|  | Yes | 988 (51%) | 668 (51%) | 320 (52%) |  |
| Baseline hemoglobin, g/dL, median (IQR) | | 12.9 (11.5-14.1) | 13 (11.6-14.2) | 12.5 (11.1-13.8) | <0.01 |
| Baseline NLR, median (IQR) |  | 2.39 (1.76-3.3) | 2.30 (1.7-3.3) | 2.48 (1.9-3.4) | 0.02 |
| Baseline CRP, mg/dL, median (IQR) | | 0.14 (0.06-0.47) | 0.14 (0.06-0.48) | 0.14 (0.06-0.44) | 0.42 |
| WHO-performance status | 0 or 1 | 1835 (96%) | 1260 (96.4%) | 575 (94.3%) | 0.04 |
|  | 2 or more | 82 (4.2%) | 47 (3.6%) | 35 (5.7%) |  |
| Clinical N category # | N0 | 1723 (90%) | 1199 (92%) | 524 (86%) | <0.01 |
|  | N1-2 | 194 (10%) | 108 (8.3%) | 86 (14%) |  |
| Pathological T category # | Ta | 274 (14%) | 157 (12%) | 117 (19%) | 0.12 |
|  | T1 | 461 (24%) | 346 (27%) | 115 (19%) |  |
|  | T2 | 309 (16%) | 217 (17%) | 92 (15%) |  |
|  | T3 | 707 (37%) | 487 (37%) | 220 (36%) |  |
|  | T4 | 92 (4.8%) | 57 (4.4%) | 35 (5.7%) |  |
|  | Tis | 74 (3.9%) | 43 (3.3%) | 31 (5.1%) |  |
| Pathological N category # | N0 | 1213 (63%) | 795 (61%) | 418 (69%) | 0.09 |
|  | N1-2 | 172 (9.0%) | 101 (7.7%) | 71 (12%) |  |
|  | Unknown ### | 532 (28%) | 411 (32%) | 121 (20%) |  |
| Tumor grade ## | Low grade | 660 (34%) | 544 (42%) | 116 (19%) | <0.01 |
|  | High grade | 1154 (60%) | 739 (57%) | 415 (68%) |  |
|  | Unknown | 104 (5.4%) | 24 (1.8%) | 80 (13%) |  |
| Carcinoma in situ | Negative | 1648 (86%) | 1139 (87%) | 509 (83%) | 0.03 |
|  | Positive | 269 (14%) | 168 (13%) | 101 (17%) |  |
| Lymphovascular invasion | Negative | 1054 (55%) | 702 (54%) | 352 (58%) | 0.33 |
|  | Positive | 703 (37%) | 452 (35%) | 251 (41%) |  |
|  | Unknown | 160 (8.3%) | 153 (12%) | 7 (1.2%) |  |
| Adjuvant chemotherapy | No | 1441 (75%) | 990 (76%) | 451 (74%) | 0.39 |
|  | Performed | 476 (25%) | 317 (24%) | 159 (26%) |  |
| UTUC, upper urinary tract cancer; RNU, radical nephrouretrectomy; IQR, interquartile range; NLR, neutrophil lymphocyte ratio; CRP, c-reactive protein; Cre, creatinine; WHO, the World Health Organization; # 2016 Union for International Cancer Control TNM Staging system; ## 2004 WHO classification; ### Including cases who did not receive lymph node dissection. | | | | | |

| **Supplementary Table 2. Multivariate Fine and Gray subdistribution hazard model for extra-urinary recurrence, cancer-specific death, and bladder recurrence in the UTUC development cohort, selected based on Akaike's Information Criterion** | | | | | | | | | | | | | | | | |  |
| --- | --- | --- | --- | --- | --- | --- | --- | --- | --- | --- | --- | --- | --- | --- | --- | --- | --- |
| Factors (Selected based on AIC) | Category |  | Extra-urinary tract recurrence | | | |  | Cancer-specific death | | | |  | Bladder recurrence | | | | |
|  |  |  | β coefficient | HR | 95% CI | *p* value |  | β coefficient | HR | 95% CI | *p* value |  | β coefficient | HR | 95% CI | *p* value | |
| Sex | Male |  |  |  |  |  |  |  |  |  |  |  |  | 1 |  |  | |
|  | Female |  |  |  |  |  |  |  |  |  |  |  | -0.41 | 0.67 | 0.49–0.90 | 0.0087 | |
| Location of main tumor | Renal pelvis |  |  | 1 |  |  |  |  | 1 |  |  |  |  | 1 |  |  | |
|  | Upper ureter |  |  | - | - | - |  |  | 1.77 | 0.98–3.20 | 0.059 |  |  | - | - | - | |
|  | Middle ureter |  | 0.38 | 1.54 | 1.00–2.36 | 0.049 |  | 0.65 | 1.98 | 1.23–3.18 | 0.0048 |  | 0.34 | 1.39 | 0.97–2.00 | 0.077 | |
|  | Lower ureter |  | 0.13 | 1.41 | 0.98–2.02 | 0.062 |  |  | - | - | - |  | 0.15 | 1.38 | 0.99–1.93 | 0.054 | |
| Multiplicity | Single |  |  |  |  |  |  |  |  |  |  |  |  | 1 |  |  | |
|  | Multiple |  |  |  |  |  |  |  |  |  |  |  | 0.44 | 1.58 | 1.16–2.17 | 0.0041 | |
| Hydronephrosis | Negative |  |  |  |  |  |  |  |  |  |  |  |  | 1 |  |  | |
|  | Positive |  |  |  |  |  |  |  |  |  |  |  | -0.35 | 0.73 | 0.55–0.96 | 0.027 | |
| Baseline hemoglobin | ≥ LLN |  |  |  |  |  |  |  |  |  |  |  |  | 1 |  |  | |
|  | < LLN |  |  |  |  |  |  |  |  |  |  |  |  | 1.24 | 0.95–1.62 | 0.11 | |
| Baseline NLR | ≤ 3.0 |  |  | 1 |  |  |  |  | 1 |  |  |  |  | 1 |  |  | |
|  | > 3.0 |  | 0.38 | 1.44 | 1.05–1.96 | 0.022 |  | 0.51 | 1.71 | 1.16–2.51 | 0.0065 |  | 0.36 | 1.33 | 1.01–1.76 | 0.043 | |
| Clinical N category | N0 |  |  | 1 |  |  |  |  | 1 |  |  |  |  |  |  |  | |
|  | N1-3 |  | 1.04 | 2.72 | 1.74–4.24 | <0.0001 |  | 1.01 | 2.02 | 1.15–3.55 | 0.015 |  |  |  |  |  | |
| Pathological T category | Ta |  |  | 1 |  |  |  |  | 1 |  |  |  |  | 1 |  |  | |
|  | Tis |  |  | - | - | - |  |  | - | - | - |  |  | - | - | - | |
|  | T1 |  | 0.61 | 1.82 | 0.82–4.01 | 0.14 |  | 0.85 | 11.9 | 1.59–89.22 | 0.016 |  |  | - | - | - | |
|  | T2 |  | 1.92 | 2.47 | 1.15–5.33 | 0.021 |  | 1.27 | 9.81 | 1.31–73.71 | 0.026 |  |  | - | - | - | |
|  | T3 |  | 2.39 | 3.83 | 1.80–8.15 | 0.0005 |  | 2.11 | 21.61 | 2.94–159.0 | 0.0025 |  | -0.27 | 0.79 | 0.60–1.05 | 0.1 | |
|  | T4 |  | 3.39 | 10.52 | 4.12–26.85 | <0.0001 |  | 3.17 | 73.41 | 9.21–585.4 | <0.0001 |  | -1.83 | 0.20 | 0.07–0.56 | 0.0022 | |
| Tumor grade (WHO 2004) | Low-grade |  |  | 1 |  |  |  |  |  |  |  |  |  |  |  |  | |
|  | High-grade |  | 0.51 | 1.87 | 1.26–2.76 | 0.0018 |  |  |  |  |  |  |  |  |  |  | |
| Lymphovascular invasion | Negative |  |  | 1 |  |  |  |  | 1 |  |  |  |  |  |  |  | |
|  | Positive |  | 1.83 | 2.32 | 161–3.35 | <0.0001 |  | 1.02 | 2.37 | 1.47–3.82 | 0.0004 |  |  |  |  |  | |
| UTUC, upper urinary tract cancer; AIC, Akaike's Information Criterion; HR, hazard ratio; CI, confidence interval; LLN, lower limit of the normal; NLR, Neutrophil lymphocyte rate; WHO, the World Health Organization; β coefficient, regression coefficient The best combination of variable subsets was selected from all the variable combinations according to the Akaike information criterion. | | | | | | | | | | | | | | | | |  |

| **Supplementary Table 3. Multivariate Fine and Gray subdistribution hazard model for Extra-urinary recurrence, cancer-specific death, and bladder recurrence in the renal pelvic urothelial carcinoma (RPUC) development cohort, selected based on Akaike's Information Criterion** | | | | | | | | | | | | | | | | |
| --- | --- | --- | --- | --- | --- | --- | --- | --- | --- | --- | --- | --- | --- | --- | --- | --- |
| Factors (Selected based on AIC) | Category |  | Extra-urinary tract recurrence | | | |  | Cancer-specific death | | | |  | Bladder recurrence | | | |
|  |  |  | β coefficient | HR | 95% CI | *P-*value |  | β coefficient | HR | 95% CI | *P*-value |  | β coefficient | HR | 95% CI | *P-*value |
| Sex | Male |  |  |  |  |  |  |  |  |  |  |  |  | 1 |  |  |
|  | Female |  |  |  |  |  |  |  |  |  |  |  | -0.54 | 0.6 | 0.39–0.89 | 0.011 |
| Multiplicity | Single |  |  |  |  |  |  |  |  |  |  |  |  | 1 |  |  |
|  | Multiple |  |  |  |  |  |  |  |  |  |  |  | 0.49 | 1.56 | 1.04–2.32 | 0.03 |
| Clinical N category | N0 |  |  | 1 |  |  |  |  | 1 |  |  |  |  |  |  |  |
|  | N1-3 |  | 2.28 | 3.63 | 2.18–6.03 | <0.0001 |  | 2.03 | 2.51 | 1.33–4.75 | 0.0048 |  |  |  |  |  |
| Pathological T category | Ta |  |  | 1 |  |  |  |  | 1 |  |  |  |  | 1 |  |  |
|  | Tis |  |  | - | - | - |  |  | - | - | - |  |  | - | - | - |
|  | T1 |  | 0.13 | 1.60 | 0.54-4.72 | 0.39 |  | 1.61 | 4.81 | 0.62–37.37 | 0.13 |  |  | - | - | - |
|  | T2 |  | 1.75 | 2.24 | 1.06–4.75 | 0.035 |  | 1.44 | 4.24 | 0.49–36.79 | 0.19 |  |  | - | - | - |
|  | T3 |  | 2.04 | 3.32 | 1.83–6.03 | <0.0001 |  | 2.40 | 10.02 | 1.40–71.97 | 0.022 |  |  | - | - | - |
|  | T4 |  | 3.88 | 8.16 | 3.67–18.12 | <0.0001 |  | 3.62 | 35.47 | 4.62–272.1 | 0.0006 |  | -1.55 | 0.12 | 0.028–0.52 | 0.0047 |
| Tumor grade (WHO 2004) | Low-grade |  |  | 1 |  |  |  |  | 1 |  |  |  |  | 1 |  |  |
|  | High-grade |  | 1.45 | 1.75 | 1.07–2.86 | 0.027 |  |  | 1.54 | 0.84–2.81 | 0.17 |  | 0.51 | 1.55 | 1.09–2.2 | 0.015 |
| Lymphovascular invasion | Negative |  |  | 1 |  |  |  |  | 1 |  |  |  |  |  |  |  |
|  | Positive |  | 1.73 | 1.82 | 1.18–2.81 | 0.0072 |  | 1.26 | 1.97 | 1.07–3.64 | 0.03 |  |  |  |  |  |
| RPUC, renal pelvic urothelial carcinoma; AIC, Akaike's Information Criterion; HR, hazard ratio; CI, confidence interval; LLN, lower limit of the normal; NLR, Neutrophil lymphocyte rate; WHO, the World Health Organization; β coefficient, regression coefficient. The best combination of variable subsets was selected from all the variable combinations according to the Akaike information criterion. | | | | | | | | | | | | | | | | |

| **Supplementary Table 4. Multivariate Fine and Gray subdistribution hazard model for Extra-urinary recurrence, cancer-specific death, and bladder recurrence in the ureteral urothelial carcinoma (UUC) cohort, selected based on Akaike's Information Criterion** | | | | | | | | | | | | | | | | |
| --- | --- | --- | --- | --- | --- | --- | --- | --- | --- | --- | --- | --- | --- | --- | --- | --- |
| Factors (Selected based on AIC) | Category |  | Extra-urinary tract recurrence | | | |  | Cancer-specific death | | | |  | Bladder recurrence | | | |
|  |  |  | β coefficient | HR | 95% CI | *P-*value |  | β coefficient | HR | 95% CI | *P*-value |  | β coefficient | HR | 95% CI | *P-*value |
| Sex | Male |  |  |  |  |  |  |  | 1 |  |  |  |  |  |  |  |
|  | Female |  |  |  |  |  |  | 0.71 | 1.99 | 1.08–3.7 | 0.029 |  |  |  |  |  |
| Multiplicity | Single |  |  |  |  |  |  |  |  |  |  |  |  | 1 |  |  |
|  | Multiple |  |  |  |  |  |  |  |  |  |  |  | 0.62 | 1.87 | 1.15–3.06 | 0.012 |
| Hydronephrosis | Negative |  |  |  |  |  |  |  |  |  |  |  |  | 1 |  |  |
|  | Positive |  |  |  |  |  |  |  |  |  |  |  | -0.45 | 0.68 | 0.45–1.02 | 0.063 |
| Baseline hemoglobin | ≥ LLN |  |  |  |  |  |  |  | 1 |  |  |  |  | 1 |  |  |
|  | < LLN |  |  |  |  |  |  | 1.95 | 3.27 | 1.71–6.22 | 0.00032 |  | 0.55 | 1.47 | 0.99–2.19 | 0.057 |
| Baseline NLR | ≤ 3.0 |  |  | 1 |  |  |  |  | 1 |  |  |  |  | 1 |  |  |
|  | > 3.0 |  | 0.63 | 1.88 | 1.19–2.99 | 0.0074 |  | 0.71 | 2.09 | 1.18–3.70 | 0.012 |  | 0.37 | 1.44 | 0.97–2.16 | 0.072 |
| Pathological T category | Ta |  |  | 1 |  |  |  |  | 1 |  |  |  |  | 1 |  |  |
|  | Tis |  |  | - | - | - |  |  | - | - | - |  |  | - | - | - |
|  | T1 |  |  | - | - | - |  |  | - | - | - |  |  | - | - | - |
|  | T2 |  | 0.53 | 1.50 | 0.58-3.9 | 0.40 |  | 0.97 | 1.40 | 0.55-3.87 | 0.50 |  |  | - | - | - |
|  | T3 |  | 1.54 | 1.60 | 0.94–2.72 | 0.084 |  | 1.88 | 2.24 | 1.21–4.15 | 0.01 |  | -0.59 | 0.61 | 0.39–0.97 | 0.037 |
|  | T4 |  | 4.49 | 10.69 | 3.10–36.88 | 0.00018 |  | 2.96 | 4.89 | 1.87–12.76 | 0.0012 |  | -0.29 | 0.68 | 0.15-3.05 | 0.62 |
| Cacrinoma in situ | Negative |  |  |  |  |  |  |  | 1 |  |  |  |  |  |  |  |
|  | Positive |  |  |  |  |  |  | -0.62 | 0.44 | 0.21–0.93 | 0.032 |  |  |  |  |  |
| Lymphovascular invasion | Negative |  |  | 1 |  |  |  |  | 1 |  |  |  |  |  |  |  |
|  | Positive |  | 2.33 | 4.02 | 2.24–7.22 | <0.0001 |  | 1.57 | 3.93 | 1.89–8.15 | 0.00024 |  |  |  |  |  |
| UUC, Ureteral urothelial carcinoma; AIC, Akaike's Information Criterion; HR, hazard ratio; CI, confidence interval; LLN, lower limit of the normal; NLR, Neutrophil lymphocyte rate; WHO, the World Health Organization; β coefficient, regression coefficient. The best combination of variable subsets was selected from all the variable combinations according to the Akaike information criterion. | | | | | | | | | | | | | | | | |

| **Supplementary Table 5. Actual probabilities at 2, 5, and 10 years in patients with UTUC stratified by the J-NICE risk models.** | | | | | | | | | | | | |
| --- | --- | --- | --- | --- | --- | --- | --- | --- | --- | --- | --- | --- |
| The J-NICE risk stratification model |  | Extra-urinary tract recurrence % Yrs (95% CI) | | |  | Cancer-specific death % Yrs (95% CI) | | |  | Intravesical recurrence % Yrs (95% CI) | | |
|  |  | 2 year | 5 year | 10 year |  | 2 year | 5 year | 10 year |  | 2 year | 5 year | 10 year |
| Overall UTUC risk model (n=1307) | | | | | | | | | | | | |
| All |  | 25.9 (23.4-28.6) | 34.0 (30.9-37.0) | 38.3 (34.6-41.9) |  | 12.4 (10.5-14.4) | 23.2 (20.5-26.0) | 29.1 (25.5-32.7) |  | 34.3 (21.4-37.1) | 43.4 (40.2-46.5) | 48.1 (44.2-51.9) |
| Low-risk |  | 5.7 (3.7-8.5) | 10.6 (7.5-14.3) | 15.7 (11.1-21.0) |  | 0.94 (0.3-2.6) | 6.6 (4.0-10.0) | 11.4 (7.2-16.6) |  | 23.7 (11.8-37.9) | 23.7 (11.8-37.9) | 23.7 (11.8-37.9) |
| Intermeditate-risk |  | 22.0 (18.1-26.2) | 31.2 (26.3-36.1) | 36.5 (30.2-42.8) |  | 7.8 (5.5-10.5) | 17.4 (13.6-21.7) | 23.8 (18.5-29.6) |  | 29.7 (25.9-33.7) | 39.1 (34.5-43.5) | 41.0 (36.0-45.8) |
| High-risk |  | 51.5 (45.5-57.3) | 64.0 (56.9-70.2) | 66.2 (58.1-73.1) |  | 27.1 (22.3-32.2) | 46.5 (40.0-52.8) | 53.9 (45.4-61.7) |  | 38.9 (34.5-43.1) | 47.9 (43.1-52.6) | 55.8 (49.3-61.7) |
| Highest-risk |  | 84.9 (65.8-93.8) | 89.9 (69.2-97.0) | 89.9 (69.2-97.0) |  | 60.2 (40.7-75.1) | 74.8 (49.1-88.8) | 74.8 (49.1-88.8) |  | 49.5 (31.4-65.4) | 67.9 (46.1-82.4) | 80.5 (48.7-93.6) |
| RPUC-specific risk model (n=766) | | | | | | | | | | | | |
| All |  | 26.9 (23.6-30.4) | 33.1 (29.3-37.0) | 37.3 (32.6-42.0) |  | 15.7 (13.1-18.6) | 24.4 (20.9-28.0) | 29.4 (25.1-33.9) |  | 31.1 (27.5-34.7) | 39.1 (35.0-43.1) | 43.4 (38.3-48.3) |
| Low-risk |  | 3.7 (1.6-7.1) | 9.6 (5.5-15.0) | 11.8 (7.0-18.0) |  | 1.7 (0.6-4.0) | 9.2 (5.7-13.7) | 12.0 (7.6-17.5) |  | 21.9 (0.9-61.2) | 21.9 (0.9-61.2) | 21.9 (0.9-61.2) |
| Intermeditate-risk |  | 19.1 (14.6-24.1) | 26.2 (20.5-32.2) | 31.2 (23.9-38.8) |  | 10.2 (6.3-15.2) | 17.4 (11.8-24.0) | 23.9 (16.5-32.0) |  | 28.1 (23.6-32.8) | 35.8 (30.5-41.1) | 39.2 (32.4-45.8) |
| High-risk |  | 54.3 (46.1-61.7) | 60.7 (51.8-68.5) | 66.0 (55.1-74.8) |  | 30.0 (23.1-37.2) | 42.6 (34.2-50.7) | 50.3 (38.4-61.1) |  | 33.4 (27.4-39.5) | 42.9 (35.8-49.8) | 50.0 (41.1-58.2) |
| Highest-risk |  | 77.6 (61.3-87.7) | 81.5 (64.5-90.9) | 81.5 (64.5-90.9) |  | 61.1 (46.5-72.9) | 69.0 (53.7-80.1) | 75.4 (56.6-86.9) |  | 47.1 (31.1-61.5) | 51.6 (34.2-66.5) | 51.6 (34.2-66.5) |
| UUC-specific risk model (n=541) | | | | | | | | | | | | |
| All |  | 23.9 (20.1-27.9) | 34.5 (29.8-39.3) | 38.7 (33.2-44.2) |  | 7.7 (5.5-10.3) | 21.7 (17.5-26.1) | 28.6 (22.8-34.6) |  | 37.2 (32.8-41.5) | 47.7 (42.8-52.4) | 53.1 (47.4-58.4) |
| Low-risk |  | 7.3 (4.5-11.0) | 12.6 (8.5-17.6) | 20.0 (13.4-27.6) |  | 0 (NA-NA) | 0 (NA-NA) | 0 (NA-NA) |  | 26.0 (15.2-38.2) | 33.8 (20.9-47.1) | 37.8 (23.4-52.1) |
| Intermeditate-risk |  | 30.8 (22.0-40.0) | 45.6 (34.7-55.8) | 45.6 (34.7-55.8) |  | 2.3 (0.9-5.0) | 9.6 (5.8-14.6) | 18.8 (10.6-28.8) |  | 26.5 (19.2-34.2) | 43.8 (34.5-52.6) | 45.8 (36.0-55.0) |
| High-risk |  | 50.5 (40.7-59.5) | 72.4 (60.2-81.4) | 72.4 (60.2-81.4) |  | 10.9 (6.4-16.7) | 40.0 (30.3-49.6) | 49.6 (37.9-60.2) |  | 43.1 (37.1-48.9) | 49.7 (43.3-55.8) | 58.9 (50.3-66.5) |
| Highest-risk |  | 100 (NA-NA) | 100 (NA-NA) | 100 (NA-NA) |  | 52.6 (33.6-68.5) | 87.7 (58.8-96.8) | 87.7 (58.8-96.8) |  | 49.0 (28.3-66.8) | 73.8 (47.1-88.5) | 73.8 (47.1-88.5) |
| UTUC, upper tract urothelial carcinoma; J-NICE, Japanese NIshinihon uro-onCology Extensive collaboration group; RPUC, renal pelvic urothelial carcinoma; UUC, ureteral urothelial carcinoma; CI, confidence interval; NA, not available; | | | | | | | | | | | | |
